# Genetic characterization of commercial spring maize germplasm in Northern China for hybrid breeding and trait improvement

**DOI:** 10.3389/fpls.2026.1872682

**Published:** 2026-07-10

**Authors:** Yuhan Song, Hongsen Nong, Zhenyang Liao, Yanyan Wang, Hongying Yu, Guohua Du, Yuxiao Chang, Cong Tan, Xingtan Zhang, Hong Lu

**Affiliations:** 1 Shenzhen Branch, Guangdong Laboratory of Lingnan Modern Agriculture, Agricultural Genomics Institute at Shenzhen, Chinese Academy of Agricultural Sciences, Shenzhen, China; 2 College of Agriculture, South China Agricultural University, Guangzhou, Guandong, China; 3 Spice and Beverage Research Institute, Chinese Academy of Tropical Agricultural Sciences, Wanning, Hainan, China; 4 State Key Laboratory of Agricultural Genomics, Key Laboratory of Genomics, Ministry of Agriculture, BGI Research, Shenzhen, China

**Keywords:** germplasm resource, identity by descent, maize, selective sweep, whole-genome resequencing

## Abstract

**Introduction:**

Commercial spring maize germplasm in northern China has been shaped by five decades of intensive breeding and the continual incorporation of elite domestic and introduced germplasm, but its genetic diversity, pedigree structure, and genomic signatures of selection remain incompletely characterized.

**Methods:**

We re-sequenced 108 representative inbred lines from China’s commercial spring maize germplasm and combined them with 14 previously published key inbred lines. Population structure, genetic diversity, identity-by-descent, selective sweeps, and haplotype-trait associations were analyzed across seven heterotic groups.

**Results:**

The 122 inbred lines represented nearly five decades of elite maize germplasm in northern China. Modern breeding materials showed reduced nucleotide diversity, with π decreasing from 1.58 × 10−3 to 1.34 × 10−3, and an increased deleterious mutation burden. We identified 275 Mb of high-impact IBD regions shared among elite germplasm, containing genes associated with flowering, plant architecture, photosynthesis, and grain quality. Selective sweep analyses detected genomic regions associated with agronomic traits including grain quality and floral organ development. Haplotype analyses further revealed natural alleles related to tassel structure, with differential allele frequencies between the SS and TSPT heterotic groups.

**Discussion:**

These findings provide a genomic resource for northern Chinese commercial maize germplasm and support future heterotic group optimization, marker-assisted breeding, and genomic selection.

## 1 Introduction

Maize (*Zea mays* L.) is one of the most important cereal crops globally, with its production surpassing that of both wheat and rice (FAOSTAT, 2024). It serves various purposes, including human consumption, livestock feed, and industrial applications. As a cross-pollinated plant, maize exemplifies the phenomenon of hybrid vigor, wherein hybrid varieties outperform their parental lines in terms of height, vigor, and productivity under field conditions (Hallauer and Carena, 2009). Consequently, nearly all commercially cultivated maize seeds are hybrids, created by crossing different homozygous inbred lines to produce F1 seeds. Hybrid vigor is a complex phenomenon influenced by multiple factors, with the genetic characteristics of superior parental lines playing a critical role in the development of high-yielding hybrids (Xiao et al., 2021).

The principle of hybridization forms the foundation of what is known as the Breeding 2.0 era (Wallace et al., 2018), wherein the genetic diversity and breeding potential of maize inbred lines are key to effective hybrid development. Understanding the genetic basis of commonly used inbred lines is essential for the development and selection of new maize varieties that are more productive, resilient, and capable of meeting the needs of a growing global population. The genetic background of these widely used lines is therefore a vital component in advancing maize breeding and ensuring future crop improvement (Shiferaw et al., 2011).

Genomic analysis, particularly resequencing, has emerged as a powerful tool to explore the genetic underpinnings of maize germplasms and is widely applied in identifying genetic loci that contribute to both domestication and productivity (Liu et al., 2020). Previous studies have revisited classic domestication genes, such as *tb1* and *tga1*, emphasizing their pivotal roles in maize domestication (Hufford et al., 2012). Additionally, the analysis of large-scale expression data and genomic scans has led to the identification of *ZmSWEET4c*, a hexose transporter gene involved in seed filling during domestication (Sosso et al., 2015). High-density genetic markers have also been instrumental in identifying functional genes like *lg1*, *lg2*, *ZmCCT*, *ZmCCoAOMT2*, *ZmBAM1d*, and *THP9*, which contribute to key traits in maize, such as plant architecture, flowering time, disease resistance, yield, and nutritional quality (Tian et al., 2011; Yang et al., 2013, Yang et al., 2017, Yang et al., 2019; Huang et al., 2022b). Furthermore, resequencing populations have facilitated the tracking of trait and genetic locus improvements across different heterotic groups over the past several decades of maize breeding (Wang et al., 2020; Li et al., 2022).

Despite substantial progress in maize population genomics, most previous studies focused on global diversity panels, domestication populations, or historical breeding materials. The genetic composition of contemporary commercial spring maize germplasm used in northern China remains insufficiently characterized. In particular, it remains unclear how long-term breeding has shaped genetic diversity, identity-by-descent patterns, selective sweeps, and favorable haplotypes across major heterotic groups. Understanding these genomic features is essential for heterotic group improvement, marker-assisted breeding, and genomic selection. A comprehensive evaluation of the genetic characteristics of elite inbred lines currently utilized in commercial maize production in China is critical for enhancing and renewing maize varieties. To this end, we collected and sequenced 108 core inbred lines that are widely employed in commercial hybridization in northern China. This panel represents major heterotic parents and encompasses significant genetic variation. We then constructed a consanguinity network and identified key pedigree hotspots using identity-by-descent (IBD) analysis. Additionally, we mapped selection signals that differ between breeding eras and heterotic groups. Finally, we explored the usage patterns of different haplotypes associated with traits such as tassel size (branch number). These findings provide valuable genomic insights into the current core parental lines and lay the foundation for the development of improved maize varieties in northern China.

## 2 Materials and methods

### 2.1 Sample collection, sequencing and variant calling

A total of 108 maize inbred lines were cultivated at the BGI-AGRO Agricultural Experiment Station during the spring season of 2022. Samples were collected and sequenced by BGI Genomics using the DNAseq PE150 platform. Quality control of the raw reads was performed using FastQC v0.12.1 (https://www.bioinformatics.babraham.ac.uk/projects/fastqc/), followed by read filtering with fastp software v1.0.1 (Chen et al., 2018) to obtain clean reads. Sequencing and read-mapping quality were assessed using samtools v1.22.1 (Danecek et al., 2021) flagstat and Qualimap v.2.3 (Okonechnikov et al., 2016) bamqc.Variant detection was conducted using the integrated commercial suite GTXv2.2 (available at: http://www.gtxlab.com/product/cat), based on the Zm-B73-REFERENCE-NAM-5.0 reference genome (https://download.maizegdb.org/Zm-B73-REFERENCE-NAM-5.0/) . Raw variants were filtered using bcftools v1.15.1 (Danecek et al., 2021) with the following criteria: QD < 2.0, FS > 60.0, MQ < 40.0, MQRankSum < -12.5, SOR > 3.0, and ReadPosRankSum < -8.0. Further filtering was performed with vcftools v0.1.16 (Danecek et al., 2011) based on minor allele frequency (MAF) >0.05 and missing rate <0.2.

### 2.2 Variation annotation

For annotating the SNP and InDel datasets, we used the ANNOVAR software (Wang et al., 2010) v.2020-06-07. The annotation process involved the following steps: (1) the ‘retrieve_seq_from_fasta.pl’ module was used to build the annotation database based on the reference genome. (2) the ‘table_annovar.pl’ module was used for variant annotation.

### 2.3 Population structure analysis

Phylogenetic trees were constructed using IQ-TREE2 v2.2.2.6 (Minh et al., 2020) based on 141,309 four-fold synonymous SNPs. The tree was generated with the following parameters: -st DNA -B 1000 -m GTR+F+R6+ASC -bnni -redo. The resulting tree was visualized and color-coded using the online tool iTOL (Letunic and Bork, 2024).

We pruned the SNPs in high levels of pair-wise LD using PLINK v1.9 (Purcell et al., 2007) with the parameter --indep-pairwise 50 10 0.2 to perform principal component analysis (PCA) and fastStructure (Raj et al., 2014) v.1.0 analysis. The ‘chooseK.py’ subroutine suggested that the optimal number of clusters was K = 7, with a model complexity of 6 and model components explaining the structure in the data as 7.

### 2.4 LD decay analysis

To estimate the linkage disequilibrium (LD), we calculated the squared correlation coefficient (r^2^) between pairs of SNPs using the PopLDdecay software (Zhang et al., 2019). The program parameters were set as ‘-MaxDist 500’ to calculate the average r^2^ between two SNPs in 500-kb windows.

### 2.5 IBD segment detection

Identity-by-descent (IBD) segments were inferred to characterize recent shared ancestry and genomic relatedness among the 122 maize accessions. The Beagle software (Browning et al., 2018) (beagle.22Jul22.46e.jar) was used to imputed the genotypic information. The LD-filtered VCF file was first split by chromosome using PLINK. To identify IBD segments shared among individuals across all 10 chromosomes, we employed the IBDSeq software (Browning and Browning, 2013). The genome was divided into 20 kb windows to identify the number of individuals sharing each segment. Segments were sorted by chromosome coordinate and summarized to calculate the extent of shared IBD regions among individuals. IBD segments with a count less than 2 or a total length under 1 Mb were excluded from the analysis. To construct the individual relationship network, pairwise shared IBD information was summarized across all chromosomes. Chromosome-specific shared IBD results were merged into a genome-wide IBD sharing matrix, in which each edge represented detectable IBD sharing between two accessions and edge weight reflected the cumulative shared IBD signal. This matrix (**Supplementary Table 5**) was subsequently used to evaluate genetic relatedness among accessions and to visualize the network structure of individual relationships, which was visualized using Gephi v0.10.1 software (Bastian et al., 2009). Regions of IBD shared by more than 85 individuals were classified as high-impact regions and visualized using the R package “Ideogram” (Hao et al., 2020). The threshold was selected based on the empirical distribution of genome-wide IBD-sharing frequencies, where 85 individuals corresponded to the upper tail of the distribution.

### 2.6 Selective sweep analysis

To detect selective sweeps during different breeding eras and across subgroups, we conducted the following analyses: (1) Genetic diversity and population differentiation index (F_st_) were calculated using a 20 kb sliding window with VCFtools. (2)XPCLR scores were calculated with a 20 kb window setting using the Python version of the XPCLR (Chen et al., 2010) software. (3) Genomic windows belonging to the top 5% (Wang et al., 2026) of the empirical distributions of FST and XP-CLR scores were considered candidate selective regions. Adjacent significant windows were merged into continuous genomic intervals using bedtools v2.31.1 (Quinlan and Hall, 2010).

Gene coordinates were obtained from the B73 RefGen_v5 genome annotation. Candidate genes were identified by intersecting merged selective sweep regions with annotated gene models using bedtools intersect. Genes showing at least 10% overlap with a selective sweep interval (-F 0.1) were retained as candidate genes. Redundant records resulting from multiple overlapping windows were removed, and the final non-redundant candidate gene set was used for downstream functional annotation and enrichment analyses.

### 2.7 Gene function and enrichment analysis

Protein-coding genes within the selective regions were extracted using the seqkit (Shen et al., 2016) tool. The target protein sequences were functionally annotated by eggNOG-mapper v2.1.9 (Cantalapiedra et al., 2021) based on COG, KOG (Tatusov et al., 2003), GO (Consortium, 2004), KEGG (Kanehisa et al., 2017), Swiss-Prot (Bairoch and Apweiler, 2000), and nr databases (Pruitt et al., 2007). Functional enrichment analysis was performed using the online tool KOBAS (Bu et al., 2021) 3.0, with a significance threshold of p < 0.05.

### 2.8 Tassel phenotyping

In May 2023, 108 maize inbred lines were planted at the A’cheng Experimental Farm in Harbin, China (45.54°N, 126.98°E). The site is located in the southeastern Songnen Plain, a representative spring maize production region characterized by fertile black soil, an annual mean temperature of 3 °C, and annual precipitation of 518 mm. The experimental materials were sown on May 3, 2023. A randomized field trial was established using two-row plots, each 4 m in length, with 65 cm row spacing and 18 plants per row, corresponding to a planting density of approximately 69,000 plants per hectare. Tassel branch number (TBN) was evaluated at the flowering stage. Five representative plants were randomly selected from each row, and the mean TBN value of 10 plants per plot was used as the phenotypic value for each inbred line in subsequent analyses.

### 2.9 Haplotype analysis and gene expression profiling

Gene haplotypes were defined using non-synonymous SNPs as the primary criteria, and synonymous SNPs as secondary. Allele status, haplotype networks, and trait associations were processed and visualized using the R package ‘geneHapR’ (Zhang et al., 2023). Differences in tassel branch number (TBN) among haplotypes were first evaluated using the Kruskal-Wallis test. When significant differences were detected, pairwise comparisons among haplotypes were performed using Dunn’s test. Individuals with heterozygous genotypes and haplotypes represented by fewer than five individuals were excluded from downstream analyses to ensure reliable statistical inference. Expression profiles of B73 tassel and ear tissues were retrieved from the public dataset GSE247571 (Sun et al., 2024). Using the MaizeNetome online platform (Feng et al., 2023), we constructed high-confidence co-expression networks for candidate TBN-associated genes.

## 3 Results

### 3.1 Genome-wide variations and population structure of elite maize inbreds

We collected 108 maize inbreds, widely utilized across China over the past five decades, and planted them for sampling in Shenzhen in 2022. Through next-generation sequencing of these 108 accessions, we generated 2.5 TB of raw data, achieving an average sequencing depth of approximately 13.3×. To enhance our understanding of maize population structure, we also incorporated publicly available resequencing data from 17 additional accessions, including 14 maize inbreds and 3 teosintes (**Supplementary Table 1**) (Chen et al., 2022). All reads were mapped to the latest B73 reference genome (v5.0). Through rigorous variant detection and filtering, we identified 11,493,528 SNPs and 1,153,268 InDels. On average, there were approximately 5.38 thousand SNPs and 0.54 thousand InDels per megabase of the genome (**Supplementary Figure 1**). We further analyzed the locations and potential functions of these variants (**Supplementary Tables 2**, **3**). Most mutations occurred in intergenic and non-coding regions, with deleterious mutations accounting for 0.93% of the total. These mutations—encompassing stop gain, stop loss, splicing, nonsynonymous SNPs, frameshift insertions, and frameshift deletions—are more likely to affect gene function. The abundant genetic variation observed across all 125 inbreds and teosintes provides a valuable resource for investigating maize population structure and functional gene discovery.

**Figure 1:**
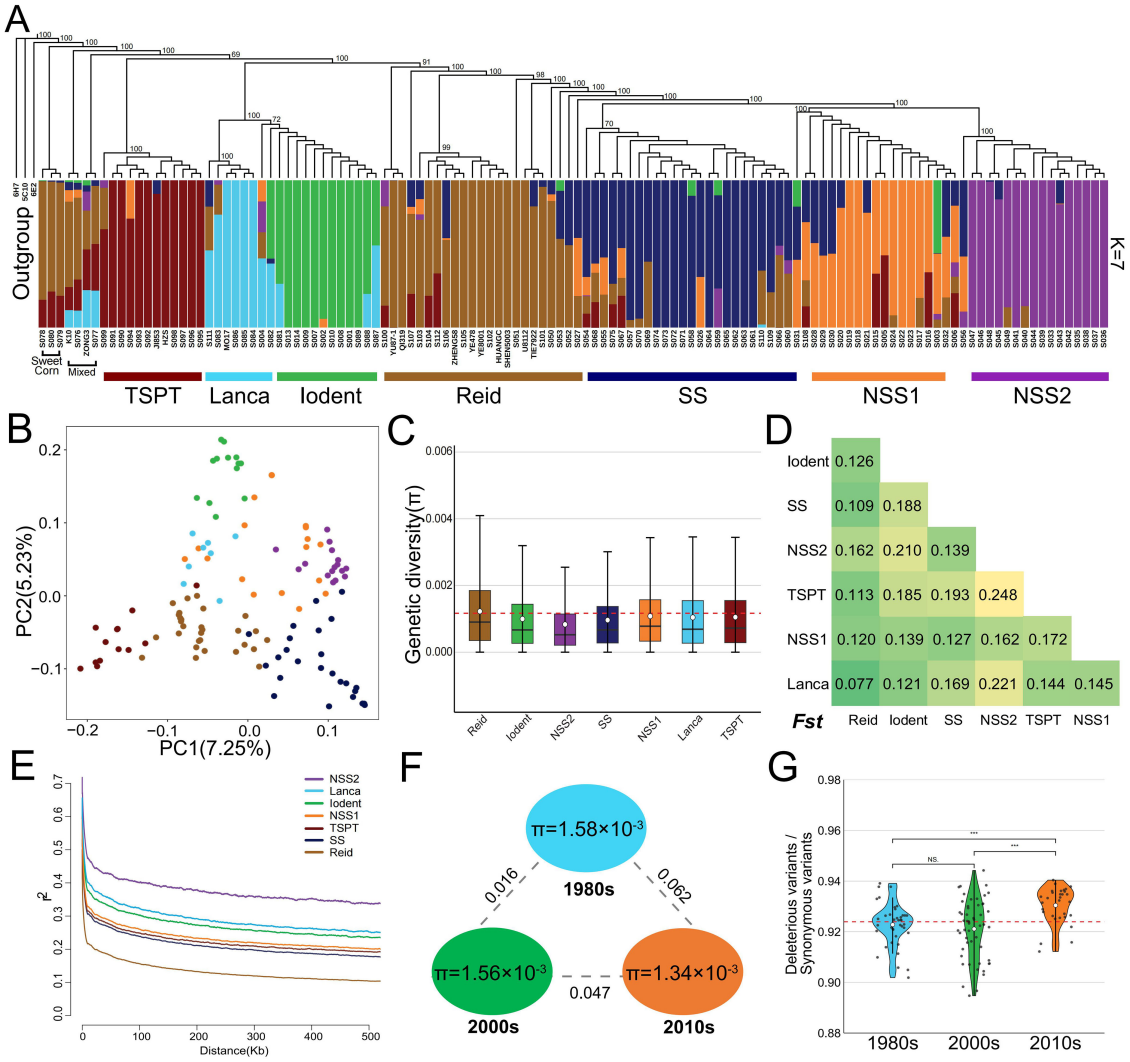
Genetic structure and diversity of 122 maize inbred lines. **(A)** Phylogenetic tree and population structure (K = 7) inferred from SNPs, with 3 teosintes used as the outgroup. Each color corresponds to a distinct population as noted. **(B)** PCA plots of maize inbreds. The first two principal components explain 7.25% and 5.23% of the genetic variance, respectively. **(C)** Genetic diversity (π) of seven heterotic groups. The red dashed line represents the overall average value (1.168×10-3). The window size is 20 Kb. **(D)** Subgroup differentiation (Fst) between heterotic groups. The heatmap displays pairwise Fst values, with higher values indicating greater genetic differentiation. **(E)** Linkage disequilibrium (LD) decay of heterotic groups within 100 Kb. The distance (Kb) of the half-maximum r2: Reid (3.9), Iodent (42.7), SS (14.1), NSS2 (202.2), TSPT (8.3), NSS1 (18.9), Lancaster (55.4). **(F)** Nucleotide diversity (π) and Fst between three breeding eras. **(G)** Normalized mutation burden (dSNP/sSNP) between three breeding eras. The P values are calculated based on pairwise t-tests, with “***” representing P values below 0.001.

To explore the genetic relationships among maize inbreds, we constructed a phylogenetic tree using 141,309 four-fold synonymous SNPs from 122 inbred lines, with three teosinte accessions as the outgroup. The phylogenetic, population structure, and principal component analyses consistently resolved seven major heterotic groups: TSPT, Lancaster, Iodent (ID), Reid, SS, NSS1, and NSS2 (**Figures 1A, B**; **Supplementary Figures 2**, **3**; **Supplementary Table 4**). These groups corresponded well with known breeding pedigrees and breeding histories. TSPT represents a traditional Chinese germplasm group, whereas Lancaster, Reid, Iodent, and SS originated primarily from temperate U.S. breeding programs. NSS1 and NSS2 represent more recently developed germplasm pools, with NSS2 derived from intensive recombination and selection within the NSS background after 2010. Collectively, these seven groups capture the major genetic foundations currently used in northern Chinese maize breeding and reflect distinct breeding histories, selection trajectories, and heterotic patterns.

### 3.2 Genetic diversity across historical breeding stages and heterosis groups

We conducted a comprehensive analysis of genetic diversity within seven maize heterotic groups. The average θπ value across all germplasm was calculated at 1.168×10^-3^, with values ranging from 0.927×10^-3^ (NSS2) to 1.453×10^-3^ (Reid) (**Figure 1C**). We further examined population differentiation coefficients, which varied from 0.0768 (between Lancaster and Reid) to 0.2480 (between TSPT and NSS2) among the different heterosis groups (**Figure 1D**). The lowest differentiation coefficient was observed between the Reid and Lancaster groups, which are part of the classic heterotic germplasm introduced from the United States during the early stages of China’s maize hybrid breeding efforts. In contrast, substantial genetic differentiation was noted between the native Chinese TSPT group and the classical American SS group (Fst = 0.193), with the greatest variance occurring in the first principal component (PC1). Furthermore, notable genetic differentiation was identified between early-stage TSPT population and the more recent NSS2 population, pointing to distinct genetic foundations that reflect the breeding history and heterotic origins of these maize inbreds.

A comparison between Chinese-bred and U.S.-introduced maize germplasm showed a slightly higher genetic diversity in the Chinese population (1.547×10^-3^) compared to the U.S. population (1.456×10^-3^) (**Supplementary Figure 4**). Except for the NSS2 population, the maximum linkage disequilibrium (LD) was observed to extend over a remarkably long distance of 202 Kb, suggesting a strong LD likely due to the short breeding history and close genetic relationships within this population. For other groups, LD decays ranged from 3.9 Kb (Reid) to 55.4 Kb (Lancaster) (**Figure 1E**; **Table 1**). Additionally, we assessed the genetic diversity of 38 early inbreds, which date back to the 1970s-1990s, alongside 67 modern inbreds, including varieties bred after 2000 (divided into the 2000–2010 and post-2010 periods). This analysis, spanning over 50 years of commercial hybridization, revealed a noticeable decrease in nucleotide diversity among modern varieties (π decreased from 1.58×10–^3^ to 1.34×10^-3^), accompanied by small Fst values between early and modern varieties (**Figure 1F**). This reduction in diversity has been particularly prominent in recent years, with an accumulation of deleterious mutations (**Figure 1G**), signaling an ongoing homogenization of maize breeding populations and narrowing of genetic diversity. Notably, genetic differentiation coefficients within heterotic groups were consistently lower than those observed between heterotic groups. In summary, the germplasm analyzed in this study includes a wide range of varieties, many of which are widely used in China. However, intensive selection over the past few decades has resulted in a reduction in the genetic diversity of breeding populations.

**Table 1:** Genetic diversity and linkage disequilibrium characteristics of seven maize heterotic groups.

Heterotic group	Nucleotide diversity (π)	Max mean r²	LD decay distance (Kb)*	Closest group (FST)	Most divergent group (FST)
Reid	1.453×10-3	0.525	3.9	Lancaster (0.077)	NSS2 (0.221)
Iodent	1.132×10-3	0.6644	43.4	Reid (0.126)	NSS2 (0.210)
SS	1.055×10-3	0.5909	14.1	Iodent (0.109)	TSPT (0.193)
NSS2	0.927×10-3	0.7344	202.2	SS (0.139)	TSPT (0.248)
TSPT	1.183×10-3	0.6122	8.5	Reid (0.113)	NSS2 (0.248)
NSS1	1.212×10-3	0.6122	19.9	Reid (0.120)	NSS2 (0.172)
Lancaster	1.21×10-3	0.6745	55.4	Reid (0.077)	NSS2 (0.221)

*LD decay distance was defined as the physical distance at which linkage disequilibrium decayed to half of its maximum value.

### 3.3 IBD segments of maize inbreeding

**Figure 2:**
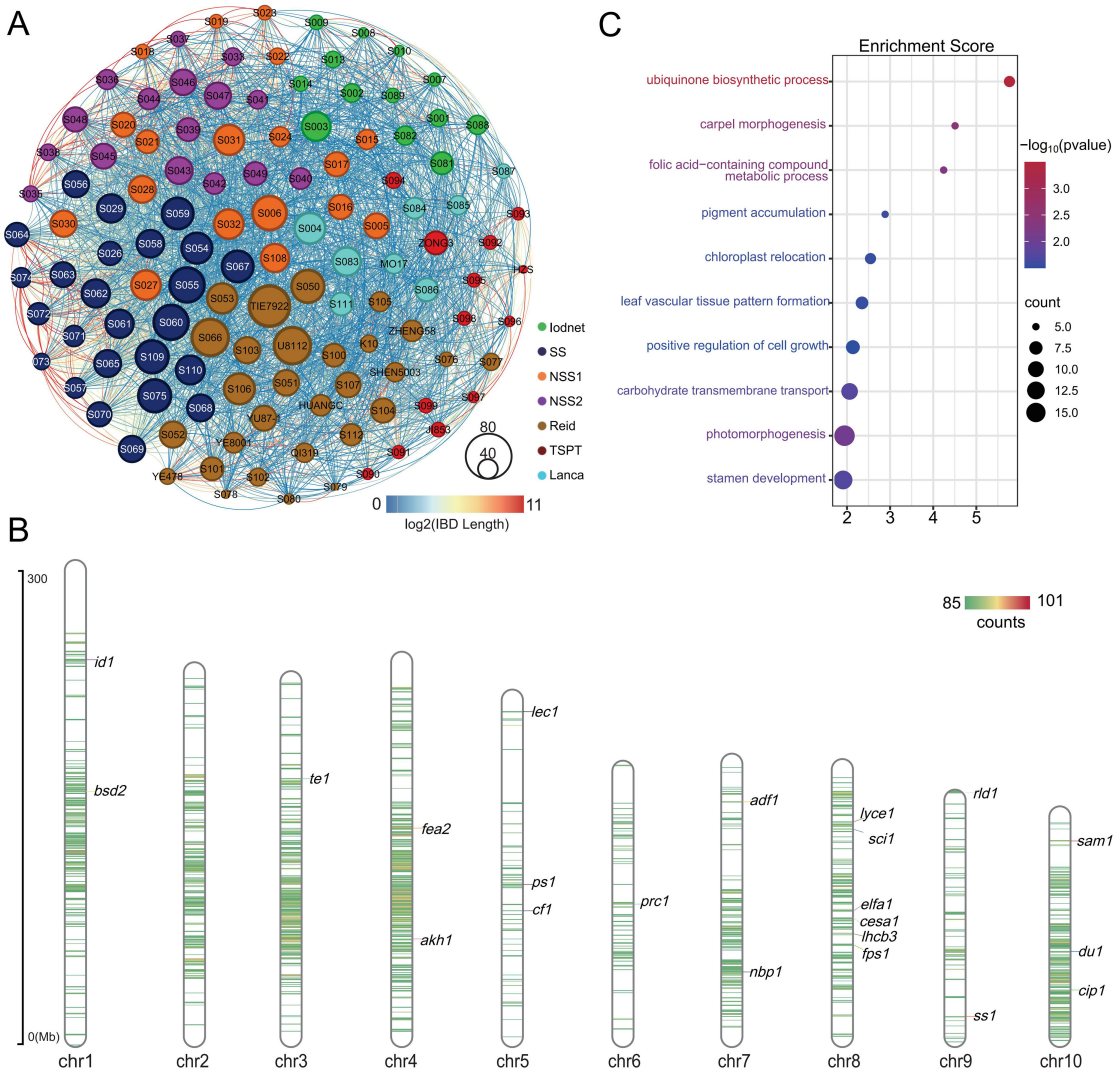
IBD segments shared in the 122 inbred lines. **(A)** inbreeding network of shared IBD by paired individuals. The size of dots represent the number of related objects. The colors of different dots represent different hybrid groups.The color of links represent the total IBD length. **(B)** Distribution of high impact IBDs (>85 individuals) on 10 chromosomes. Color scale represents the number of shared individuals. **(C)** The GO enrichment of genes covered by high impact IBDs.

We identified 133,964 pairwise IBD regions across 122 maize inbreds, with an average IBD region length of 1.75 Mb (**Supplementary Table 5**). The inbreeding network, based on pairwise IBD relationships, revealed the prominent role of the Reid and SS populations in China’s spring-sown regions, with TIE7922 and U8112, in particular, forming the most extensive connections to other individuals. In the native TSPT group, the ZONG3 line showed stronger genetic links to other groups (**Figure 2A**). IBD regions shared by more than 85 individuals (**Supplementary Figure 5**) were classified as high-impact regions (HIRs), totaling 275 Mb of sequence (average length 170 Kb). Notably, chromosomes 3 (46.6 Mb), 4 (58 Mb), and 10 (35 Mb) accounted for more than half of the total HIR (**Figure 2B**). Within these regions, 2,878 genes were located, and Gene Ontology (GO) annotations indicated their involvement in critical processes such as ubiquinone biosynthesis, carpel morphogenesis, pigment accumulation, and leaf vascular tissue pattern formation (**Figure 2C**). Several functional genes were identified within the HIRs (**Supplementary Table 6**), including *bsd2* (Brutnell et al., 1999), *cf1* (Huang et al., 2009), and *lhcb3* (Damkjær et al., 2009), which are associated with photosynthesis and respiration. Additionally, genes like *id1* (Colasanti et al., 1998), *te1* (Veit et al., 1998), *fea2* (Taguchi-Shiobara et al., 2001), *cesa1*, and *rld1* (Juarez et al., 2004a) play crucial roles in regulating reproductive development and plant morphology in maize. Furthermore, genes such as *du1* (Gao et al., 1998) and *ss1* (Knight et al., 1998) are involved in endosperm starch structure and composition, while *ps1* (Singh et al., 2003) and *lyce1* (Harjes et al., 2008) are related to carotenoid accumulation in seeds. The extensive sharing of these genomic regions across elite inbred lines suggests that they represent breeding hotspots repeatedly retained during modern hybrid development. Many HIR-associated genes are involved in flowering transition, inflorescence development, photosynthetic efficiency, and grain quality, indicating that sustained selection for adaptation, yield formation, and seed quality has shaped the genomic architecture of northern Chinese maize germplasm.

### 3.4 Genomic selection signatures in maize breeding eras

**Figure 3:**
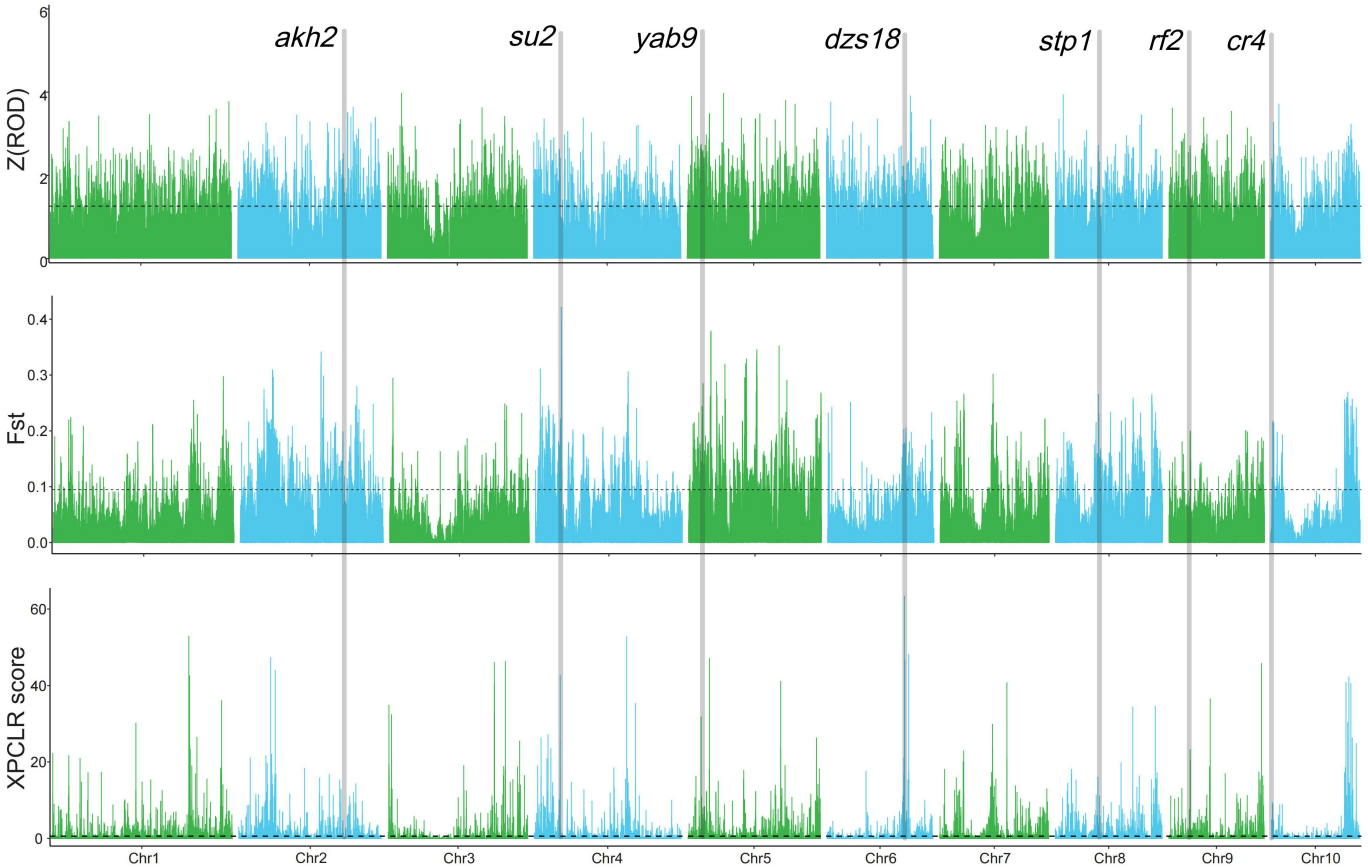
Genome-wide selective regions during modern maize breeding (1970s-1990s and 2000s). The normalized ratio of diversity(top), Fst-based genetic differentiation (middle), and XPCLR scores (bottom). Each plot uses a 20 Kb window size. Highlighted genes indicate reported functional genes located within selective sweep regions.

To identify genomic selection signals in maize from different breeding eras—specifically the early period (1970s-1990s) and the recent period (2000s)—we employed two complementary approaches: diversity (F_st_, ratio of diversity) and allele frequency differentiation (XPCLR). Our analysis focused on the top 5% of regions exhibiting the most pronounced selection signatures (**Figure 3**). A total of 504 candidate genes were identified within overlapping selective sweep regions spanning 17.10 Mb of the maize genome.(**Supplementary Table 7**). Subsequent annotation and enrichment analysis revealed key biological processes that are likely central to maize breeding. Notable GO terms include response to fructose (GO:0009750), response to sucrose (GO:0009744), floral organ senescence (GO:0080187), inflorescence development (GO:0010229), response to cytokinin (GO:0009735), and response to ethylene (GO:0009723) (**Supplementary Table 8**). These findings suggest that genes subject to selection over time are involved in critical processes such as inflorescence development, carbohydrate regulation, and plant hormone responses.

Several genes linked to key agronomic traits were identified through clear selective sweeps. For example, the *akh2* gene (Zm00001eb097580, Chr2:177327182-177347485), located on chromosome 2, encodes a bifunctional aspartokinase/homoserine dehydrogenase that regulates the Asp-derived amino acid metabolic pathway, influencing maize germination efficiency and early growth post-germination (Anzala et al., 2006). Another important gene, *su1* (Zm00001eb174590, chr4:43428507-43440253) on chromosome 4, an early-cloned starch debranching enzyme gene, has mutations leading to the sweetcorn phenotype (James et al., 1995). The *yab9* gene (Zm00001eb220660, chr5: 24518344-24528890) on chromosome 5 encodes a C2C2-YABBY transcription factor, which is involved in the development of plant leaves and floral organs by regulating gene expression (Juarez et al., 2004b). On chromosome 6, the *dzs18* (Zm00001eb281380, chr6:132167483-132168383) encodes a storage protein (zein), a major component of maize seed proteins, crucial for seed development and storage protein accumulation (Woo et al., 2001). The *stp1* (Zm00001eb344570, chr8: 75294455-75303552) on chromosome 8 encodes a sucrose transporter protein, facilitating sucrose transport within the plant (Keith et al., 1993). The *rf2* (Zm00001eb380260, chr9:36441093-36454140) on chromosome 9, a pivotal fertility restoration gene in maize research, encodes an aldehyde dehydrogenase. This gene restores male fertility in cytoplasmic male sterile (CMS-T) maize by maintaining normal pollen development through mitochondrial function and energy metabolism (Cui et al., 1996). Also, the *cr4* (crinkly4, Zm00001eb406780, chr10: 5165744-5173304) encodes a receptor kinase involved in the differentiation and development of epidermal cells, essential for the normal formation of leaves and seed coats (Becraft et al., 1996). These genes, each influencing various aspects of maize growth, reproduction, and nutrition, have been subjected to continuous artificial selection. Moreover, genome-wide selective sweep analysis proves to be a powerful tool for identifying important breeding genes in maize.

### 3.5 Genomic differentiation and functional signatures in maize heterosis groups

**Figure 4:**
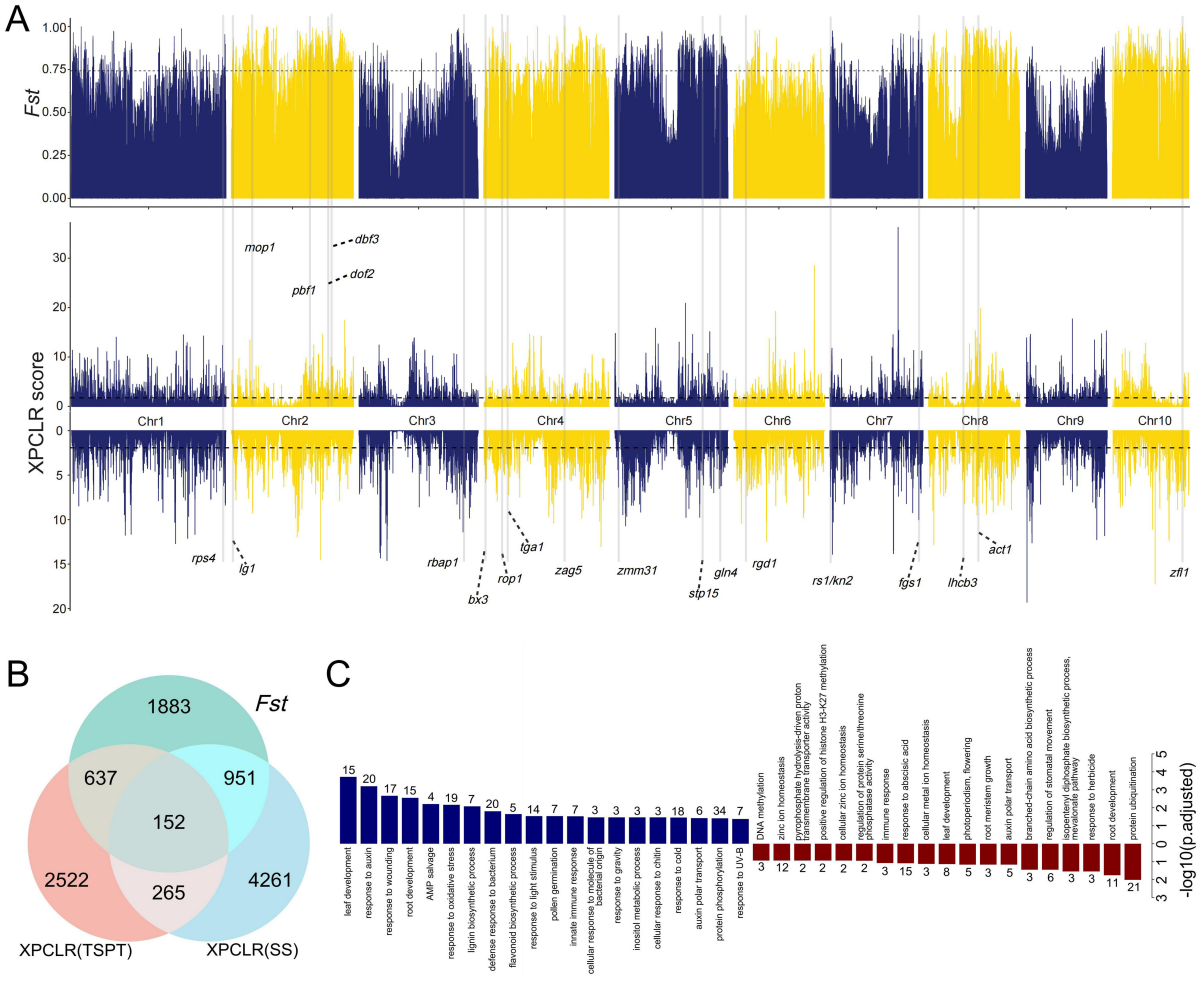
Genomic differentiation between SS and TSPT groups. **(A)** Genomic differential regions. Fst-based genetic differentiation(top), XPCLR scores of TSPT population(middle) and XPCLR scores of SS population(bottom). Each plot uses a 20 Kb window size. **(B)** The overlap of selective sweep regions identified by Fst, XP-CLR in TSPT, and XP-CLR in SS. **(C)** Gene ontology (GO) enrichment analysis of genes within the differential regions. SS population (blue) and TSPT population (red). Highlighted genes indicate reported functional genes located within selective sweep regions.

The SS population represents a widely used American maize germplasm in Northeast China, while the TSPT population, developed over several decades in China, serves as a key breeding material for the Huang-Huai region. Our analysis revealed significant genetic differentiation between these two populations, with the greatest divergence observed on PC1, which reflects the genetic distinctions between Chinese and American maize germplasm. By applying a combined Fst and XP-CLR approach, we were able to identify 1,081 distinct genomic regions differentiating the two groups (**Figure 4A**). These regions span a total of 22.95 Mb, with 446 regions in TSPT and 635 regions in SS (**Supplementary Tables 9**, **10**). Among these, 637 genes were preferentially selected in TSPT, while 951 genes were selected in SS (**Figure 4B**).

GO enrichment analysis revealed that genes selected in TSPT are involved in processes such as protein ubiquitination (GO:0016567), root development (GO:0048364), herbicide response (GO:0009635), regulation of stomatal movement (GO:0010119), photoperiodism and flowering (GO:0048573), response to abscisic acid (GO:0009737), and immune response (GO:0006955) (**Figure 4C**; **Supplementary Table 11**). Specifically, on chromosome 2 of maize, *mop1* (Zm00001eb080370, chr2:42149159-42155248) encodes an RNA-dependent RNA polymerase crucial for the establishment and maintenance of paramutation, essential for activating silenced Mutator elements and influencing recombination frequency (Alleman et al., 2006). The *pbf1* gene (Zm00001eb094330, chr2:158084097-158087728) encodes a prolamin-box binding factor that regulates storage protein gene expression during seed development (Vicente-Carbajosa et al., 1997). The *dof2* gene (Zm00001eb101420, chr2:193795034-193796605) encodes a transcription factor involved in carbon metabolism and photosynthesis, specifically affecting genes linked to the C4 photosynthetic pathway (Yanagisawa, 2000). These loci highlight the genetic basis for photosynthetic product synthesis, storage, and environmental adaptability in the TSPT population.

In contrast, genes uniquely selected in the SS population are enriched in pathways related to leaf development (GO:0048366), root development (GO:0048364), pollen germination (GO:0009846), auxin response (GO:0009733), lignin biosynthesis (GO:0009809), light response (GO:0009416), and cold stress response (GO:0009409) (**Figure 4C**; **Supplementary Table 12**). Notable genes in the SS set include *lg1* (Zm00001eb067740, chr2:4493424-4497434), which is essential for ligule and auricle development (Moreno et al., 1997), and *bx3* (Zm00001eb165550, chr4:3850064-3852826), which encodes a cytochrome P450 monooxygenase involved in the biosynthesis of the benzoxazinoid DIMBOA, providing resistance against insects, fungi, and bacteria, and offering allelopathic property. The *orp1* (Zm00001eb173100, chr4:37934555-37938833) encodes the B subunit of tryptophan synthase; mutants exhibit orange kernels and seedling lethality (Wright et al., 1992). Another significant gene in this group is *tga1* (Zm00001eb175150, chr4:46647932-46652896), a key domestication gene (Wang et al., 2005). The gene *gln4* (Zm00001eb253820, chr5:213469593-213473117) encodes glutamine synthetase, which plays a critical role in nitrogen metabolism and grain production (Martin et al., 2006). The *rgd1/lbl1* gene (Zm00001eb264310, chr6:27065686-27072113) is involved in seed and seedling development (Timmermans et al., 1998). On chromosome 7, the *rs1/kn2* gene (Zm00001eb299420, chr7:3781684-3787877) encodes a homeobox transcription factor that regulates leaf morphology and meristem development, influencing leaf shape and growth patterns (Schneeberger et al., 1998). The *fgs1* gene (Zm00001eb329710, chr7:180078246-180096892) encodes ferredoxin-dependent glutamate synthase, a key enzyme in nitrogen assimilation, which impacts protein levels within cells (Sakakibara et al., 1992). These results collectively highlight the roles of these genes in leaf development, plant morphology, nitrogen metabolism, and reproductive structures, marking key differences between SS and TSPT inbred lines.

### 3.6 Natural alleles linked to tassel morphology

**Figure 5:**
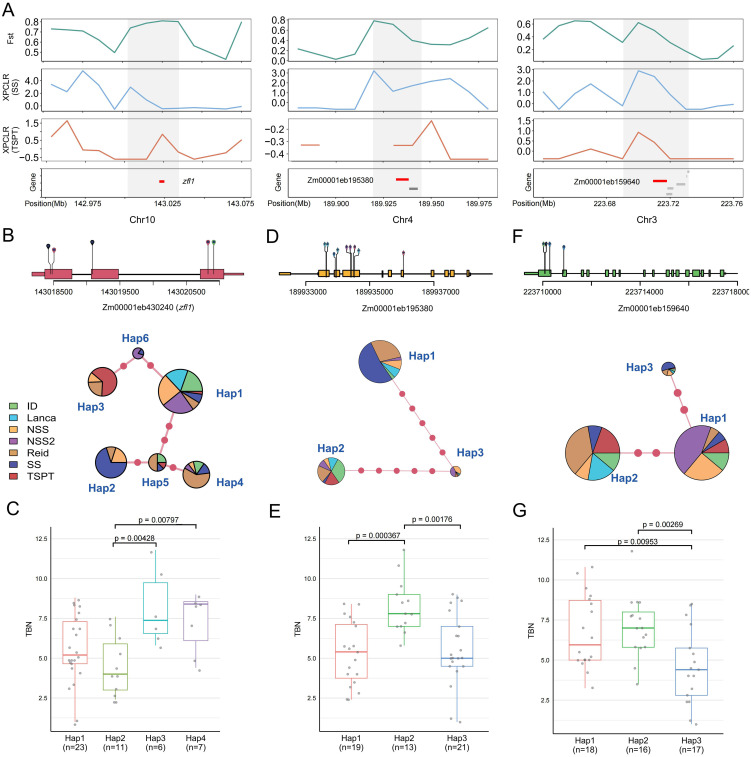
Population genetic differentiation and haplotype association analysis of three candidate gene regions. **(A)** Genome-wide scans showing Fst (green curve), XPCLR score for the SS group (blue curve), and XPCLR score for the TSPT group (red curve) in target regions on chromosomes 10 (left), 4 (middle), and 3 (right). The gray shaded areas highlight candidate gene regions. **(B, D, F)** Gene structure and haplotype networks for three candidate genes. Pie chart size indicates the frequency of each haplotype, and different colors distinguish maize populations. **(C, E, G)** Box plots of haplotype-phenotype associations. P-values derived from Dunn’s test.

Tassel branch number (TBN) is a critical trait for selecting parent lines in maize hybridization. TBN exhibited substantial phenotypic variation, ranging from 1.0 to 11.8 branches and showing significant differences among heterotic groups (**Supplementary Figures 6A, B**), indicating abundant genetic variation for tassel architecture. We identified a group of candidate genes within differentiated genomic regions potentially associated with TBN (**Figure 5A**; **Supplementary Figure 7**). Among these, the *zfl1* gene (Zm00001eb430240, Fst = 0.78, XPCLR in SS = 2.97), located within a selective sweep region (**Figure 5A**), represents the maize homolog of FLORICAULA/LEAFY (Bomblies et al., 2003). This gene encodes a key transcription factor regulating floral development and the transition from vegetative to reproductive phases. Haplotype analysis, defined by five nonsynonymous SNPs, revealed six distinct haplotypes. Hap2 dominated the SS population, while Hap3 was prevalent in TSPT lines (**Figure 5B**). Significant TBN variation was observed among major haplotypes, with Hap2 conferring the fewest branches and Hap3 exhibiting significantly higher TBN compared to Hap2 (**Figure 5C**). Two additional genes associated with TBN were identified: Zm00001eb195380 (Fst = 0.78, XPCLR in SS = 3.22) on chromosome 4 and Zm00001eb159640 (Fst = 0.65, XPCLR in SS = 2.89) on chromosome 3, both showing differential alleles among hybrid groups. Zm00001eb195380 encodes a bromo-adjacent homology (BAH) domain-containing protein, an ANTI-SILENCING 1 homolog, involved in CHG methylation within gene bodies in Arabidopsis thaliana, and regulates the floral transition (Wang and Eulgem, 2023). Haplotype networks for this gene showed Hap2-associated lines displaying significantly higher TBN than other haplotypes (**Figures 5D–G**). Zm00001eb159640 encodes the chloroplast/mitochondrial ATP-dependent zinc metalloprotease *ZmFTSH11*, which influences chloroplast development and thermotolerance (Wagner et al., 2016). Its haplotypes were associated with reduced TBN, and expression levels increased progressively during tassel and ear development (**Supplementary Figure 8A**). Further analyses implicated additional genes—Zm00001eb194930 (LRR receptor-like serine/threonine-protein kinase), Zm00001eb352910 (ktf1, KOW domain-containing transcription factor), Zm00001eb429430 (glpx7, glutathione peroxidase 7), and Zm00001eb429900 (WEB family protein)—in TBN variation, with significant haplotype-specific effects (**Supplementary Figure 7**). Public transcriptome indicates that these seven genes are dynamically expressed across tassel and ear developmental stages, exhibiting similar expression patterns (**Supplementary Figure 8A**). Co-expression network analysis demonstrated high-confidence interactions among candidate genes (**Supplementary Figure 8B**), suggesting their coordinated roles in regulating branching architecture.

## 4 Discussion

The genetic composition of elite breeding germplasm largely determines the efficiency of future hybrid development. By characterizing the genomic architecture of 108 representative commercial spring maize inbred lines, this study provides insights into how long-term breeding, germplasm exchange, and recurrent selection have shaped the current breeding resources used in northern China. Compared with previous studies focused on global diversity panels or historical germplasm collections (Wang et al., 2020; Li et al., 2022), our study specifically targets contemporary commercial parental lines that directly contribute to current hybrid production, thereby providing a practical genomic resource for breeding applications.

The reduction in nucleotide diversity observed in modern breeding materials is consistent with patterns reported in rice, wheat, and sorghum, where recurrent selection and intensive utilization of elite germplasm have narrowed the genetic base of breeding populations (Mace et al., 2013; Pont et al., 2019; Huang et al., 2022a). In northern Chinese maize breeding, recurrent recycling of successful parental lines has accelerated genetic gain but has simultaneously increased genomic similarity among breeding materials. This trend is particularly evident in NSS2, which exhibited the lowest nucleotide diversity and the slowest LD decay among all heterotic groups. In contrast, Reid maintained relatively high diversity and rapid LD decay, reflecting its broader founder base and longer breeding history. These findings suggest that future breeding efforts may benefit from introducing genetically diverse germplasm, particularly tropical and exotic materials (Fu et al., 2013; Yang et al., 2019; Chen et al., 2022), to mitigate diversity erosion and enhance long-term genetic gain.

IBD analysis provides a powerful framework for tracing founder contributions and identifying genomic regions that have been repeatedly retained during breeding. The prominent network positions of TIE7922 and U8112 reflect their extensive contributions to modern spring maize breeding programs in northern China. Both TIE7922 and U8112 were second cycle lines developed from a US hybrid PIO3382. They were among the most important elite parental lines in spring-sown maize breeding. There are more than 40 elite inbreds and 60 commercial hybrids derived from TIE7922. Here are some representative commercial hybrids developed from TIE7922: Tie12, Tie15, Tie18, Danyu26, Danyu39, Dongdan13, and Dongdan60 (Zhang et al., 2006). More importantly, the identification of 275 Mb of highly shared IBD regions suggests that a substantial proportion of the modern breeding genome has been conserved through decades of artificial selection. Genes located within these regions are enriched for flowering regulation, inflorescence development, photosynthesis, and grain quality, indicating that these biological processes have been recurrent targets of selection. The presence of key developmental regulators such as *indeterminate growth 1 (id1)*, *terminal ear 1 (te1)*, and *zea floricaula/leafy1 (zfl1)* further supports the importance of flowering transition and reproductive architecture in the adaptation and productivity of temperate maize germplasm.

Additionally, the strong genomic differentiation observed between the SS and TSPT groups reflects distinct breeding objectives and historical origins. SS represents classic American germplasm, whereas TSPT consists of locally developed parental lines selected over 60 years. In hybridization practices, SS is typically used as the female parent, while TSPT can serve as either the male or female parent. The contrasting allele frequencies observed at zfl1 and other tassel-associated loci suggest that inflorescence architecture has been subjected to different selection pressures in the two groups. Given the importance of tassel traits for pollen production, hybrid seed production, and resource allocation (Uribelarrea et al., 2002), these alleles may provide useful targets for marker-assisted breeding and heterotic group optimization.

Several limitations should be acknowledged. First, the germplasm analyzed primarily represents commercial spring maize breeding materials from northern China and does not fully capture the diversity present in tropical, subtropical, or southwestern Chinese maize populations. Second, selective sweep and haplotype analyses identify candidate genomic regions associated with breeding history and phenotypic variation but do not provide direct functional validation. Third, the phenotypic analyses focused mainly on tassel branch number, whereas many agronomically important traits remain unexplored. Future studies integrating broader germplasm resources, multi-environment phenotyping, and functional genomics approaches will provide a more comprehensive understanding of the genetic basis of maize improvement.

## 5 Conclusion

This study establishes a comprehensive genomic resource for contemporary commercial spring maize germplasm in northern China and provides insights into how long-term breeding has shaped genetic diversity, founder contributions, and favorable alleles across major heterotic groups. By integrating population genomics, IBD analysis, selective sweep detection, and haplotype analysis, we identify genomic regions and candidate alleles with potential value for marker-assisted breeding, genomic selection, and heterotic group optimization. Future integration of transcriptomic, proteomic, epigenomic, and functional genomic datasets will further improve the biological interpretation of candidate regions and accelerate precision breeding in maize.

## Data Availability

The datasets presented in this study can be found in online repositories. The names of the repository/repositories and accession number(s) can be found in the article/**Supplementary Material**. GeneBank DataBase (CNGBdb) with accession number CNP0006166; The final filtered marker matrix used in this study has been deposited in the public Zenodo repository and is freely available under DOI: 10.5281/zenodo.20421658.
