# Supplementary material for: Genetic characterization of commercial spring maize germplasm in Northern China for hybrid breeding and trait improvement
Source: Front Plant Sci. 2026 Jul 10;17:1872682. doi: 10.3389/fpls.2026.1872682 (PMC13396026; doi:10.3389/fpls.2026.1872682)
Supplement: Supplementary file 2 [file DataSheet2.docx]

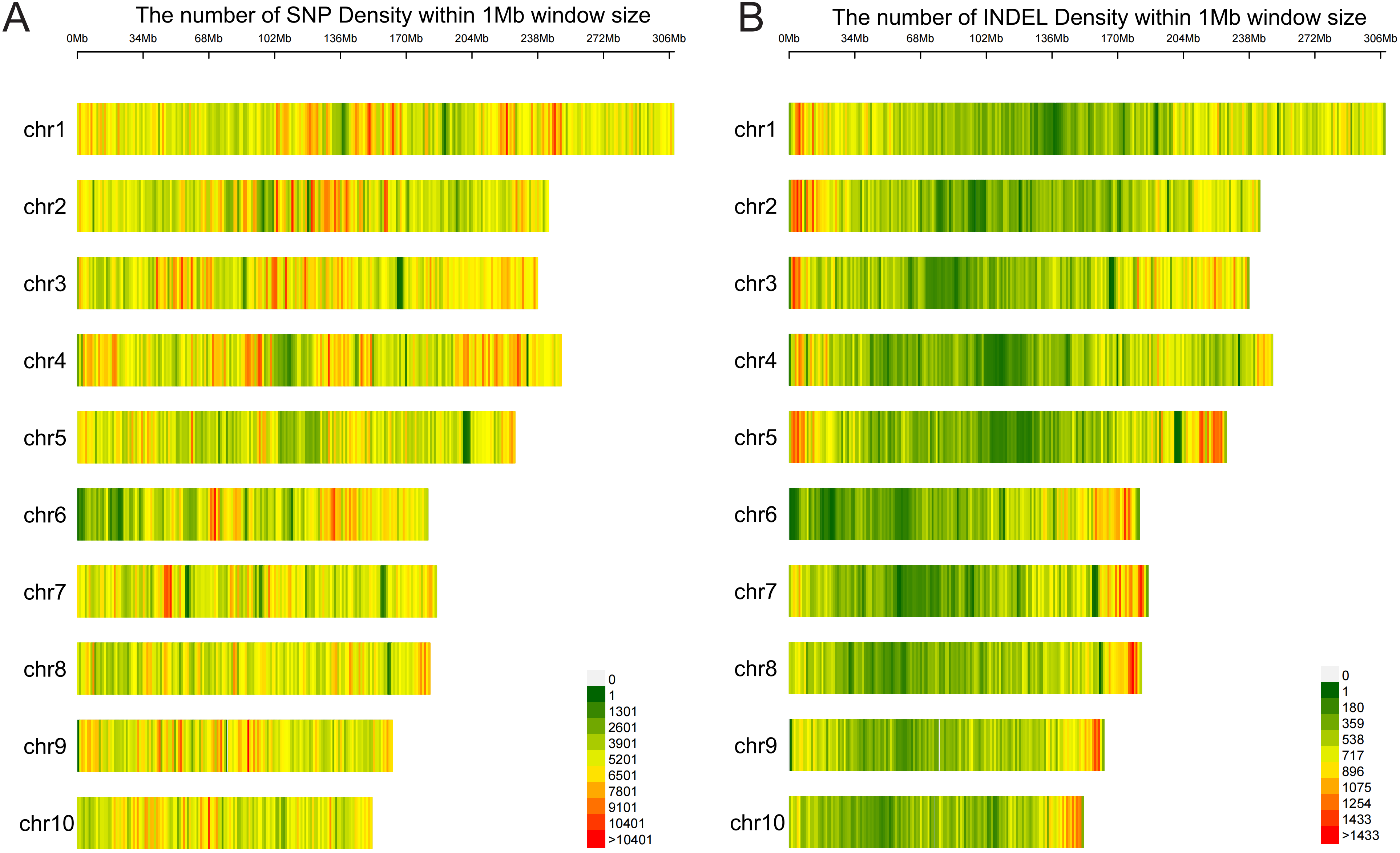


**Figure S1. Whole genome variation density within 1 Mb window size.**

SNP density(A) and InDel density(B).


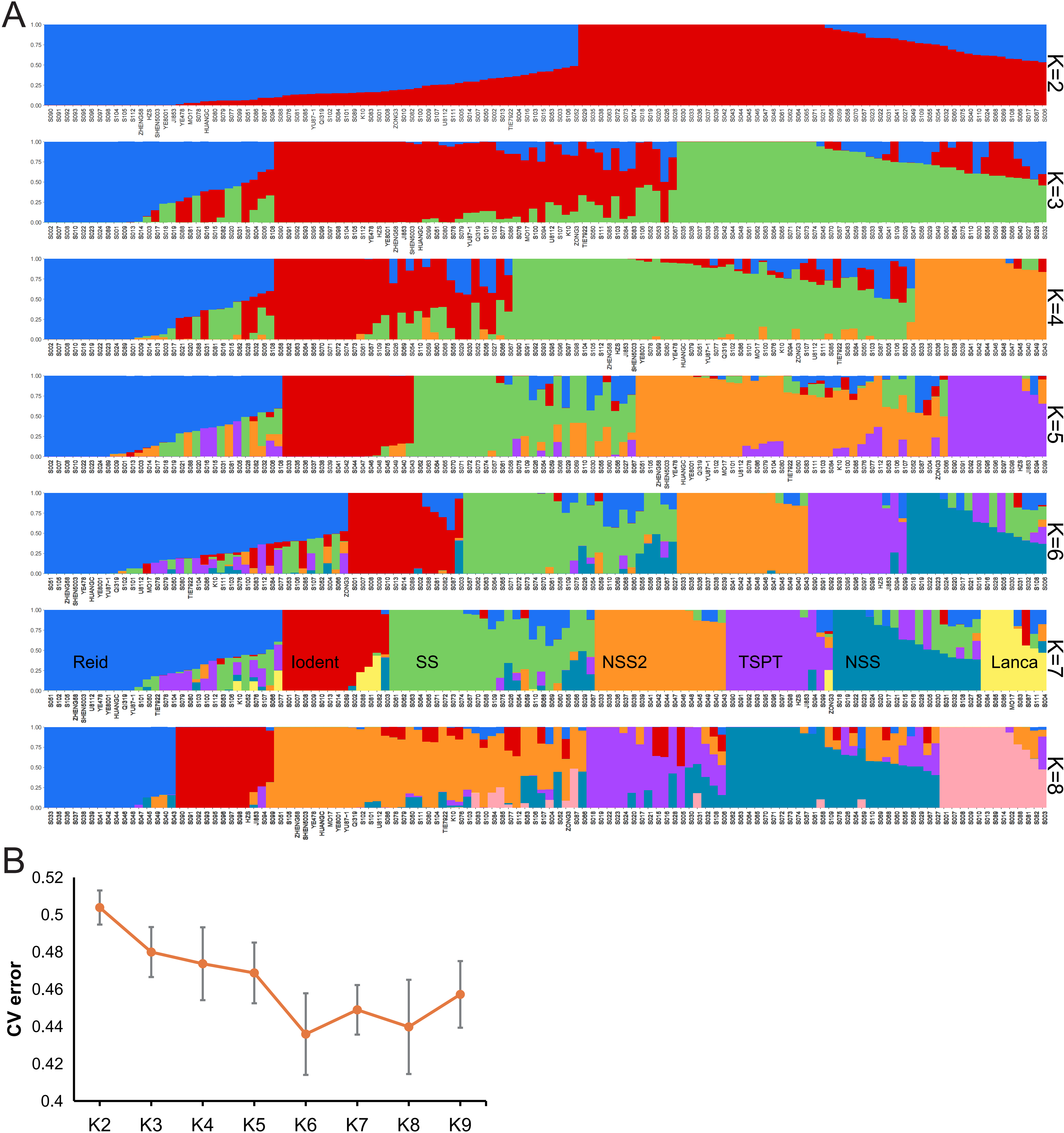


**Figure S2. Population structure(A) and cross validation plot(B) of 121 maize inbreds (From K=2 to K =9).**


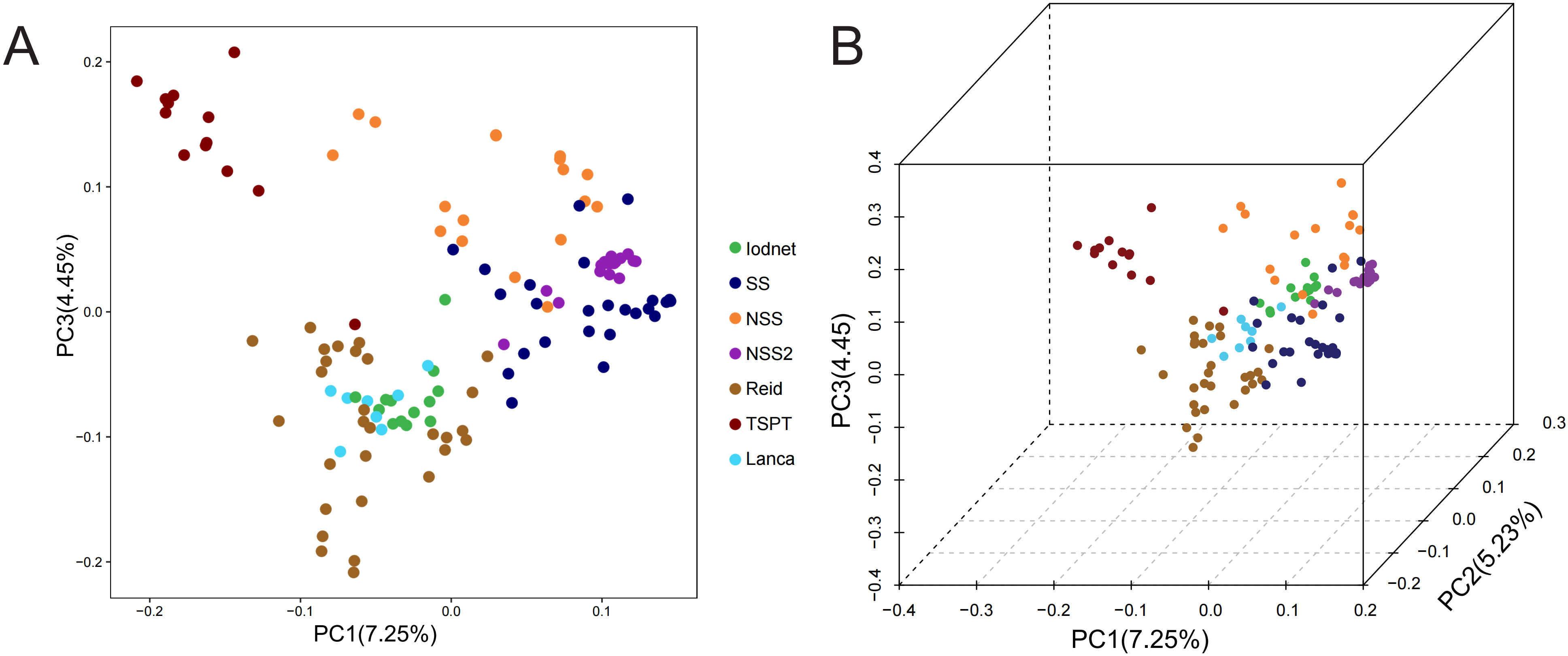


**Figure S3. The top three principal components of maize populations.**


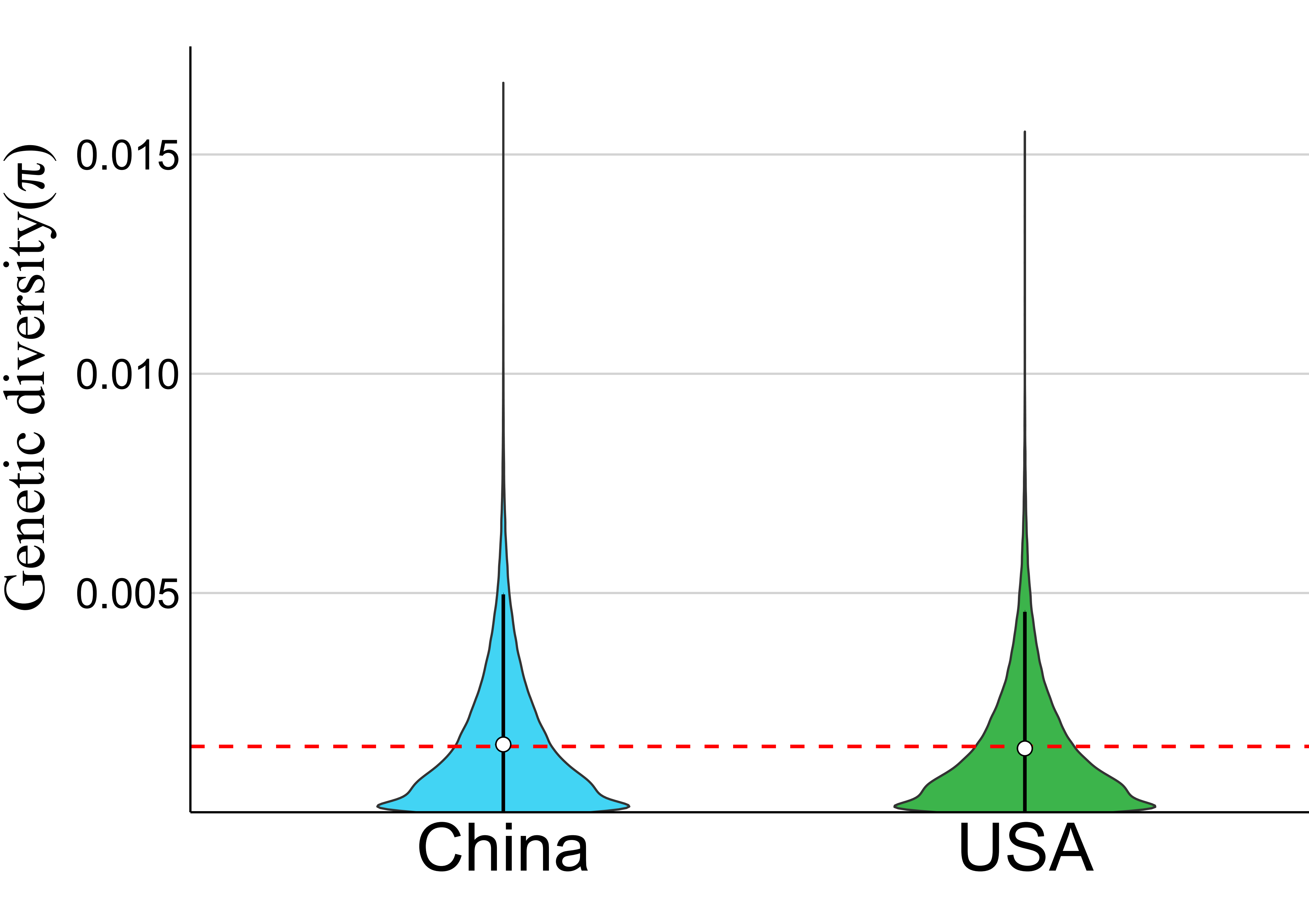


**Figure S4. Violin chart of genetic diversity of maize varieties in china and the united states.**

The red dashed line represents the average value(1.50×10^-3^).


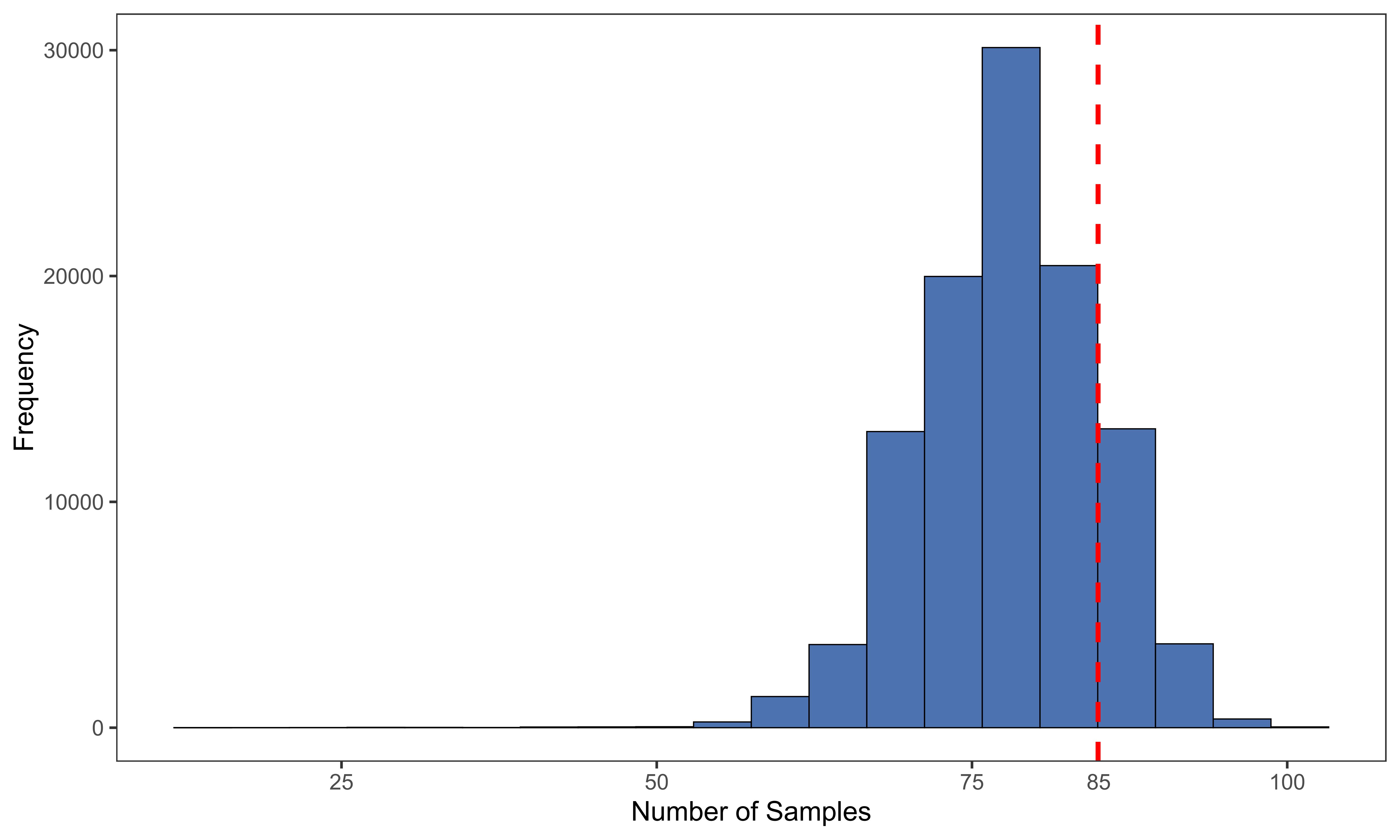


**Figure S5. Distribution of the number of individuals sharing IBD segments across genomic bins.**


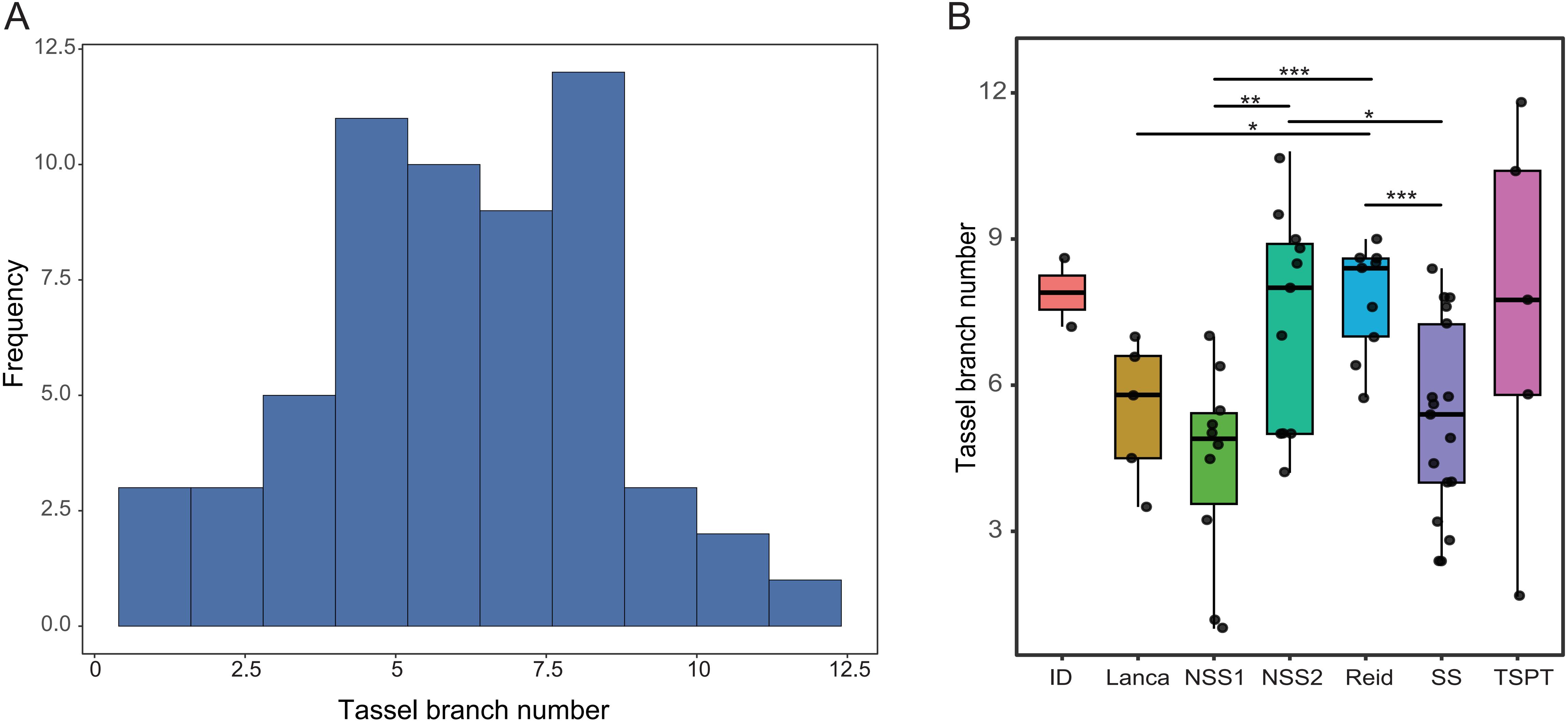


**Figure S6. Distribution of tassel branch number (TBN) in maize inbred lines.**

(A) Frequency distribution of TBN across all maize inbred lines. (B) Comparison of TBN among major heterotic groups. Each point represents an individual inbred line. Significant differences among groups were evaluated using the Kruskal–Wallis test followed by pairwise Wilcoxon rank-sum tests. Asterisks indicate significant differences (P < 0.05, P < 0.01, P < 0.001).


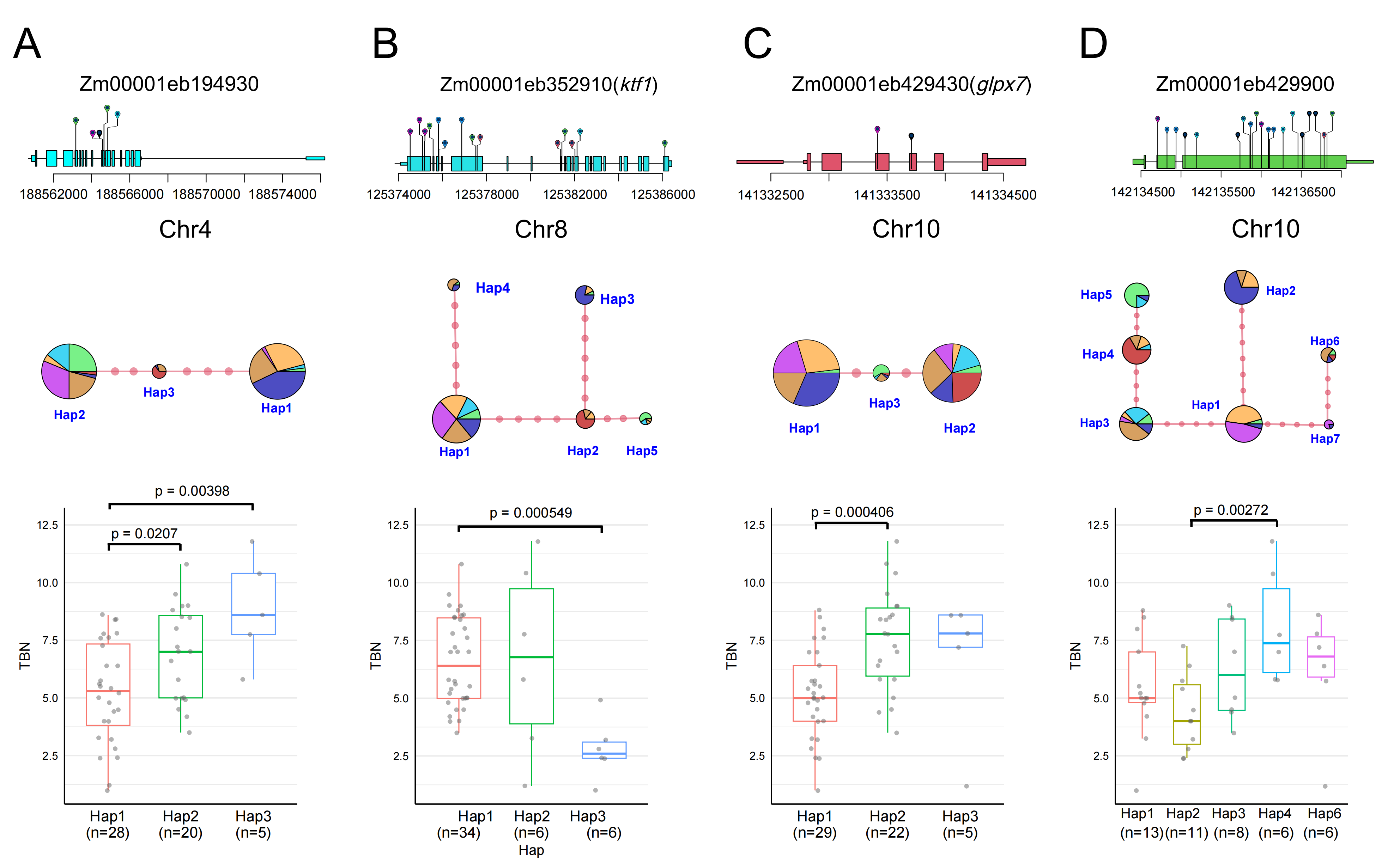


**Figure S7. Gene structure and haplotype association analysis of four candidate genes.** Pie chart size indicates the frequency of each haplotype, and different colors distinguish maize populations. P-values derived from Dunn’s test.


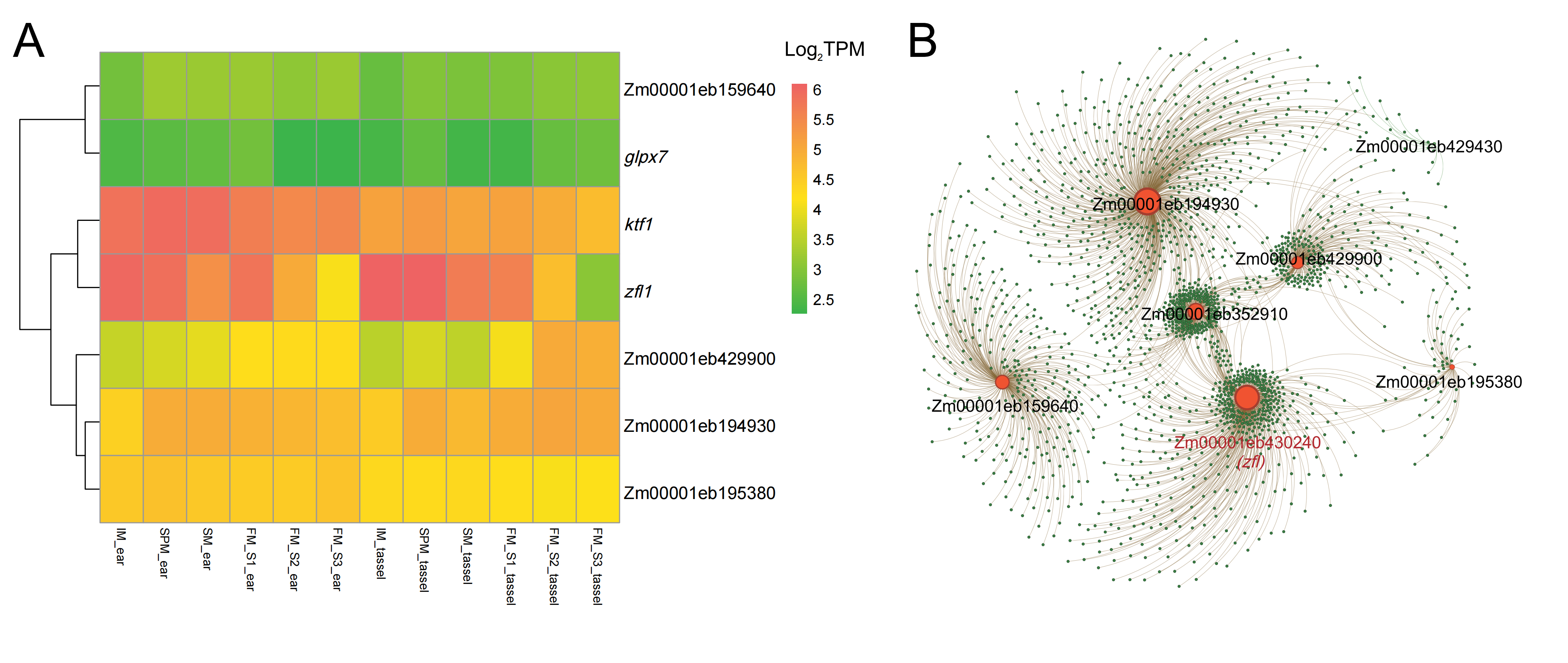


**Figure S8. Expression heatmap and co expression network of candidate genes**

1. Expression heatmap of candidate genes associated with tassel branch number across the different meristems and developmental stages. IM, inflorescence meristem; SPM, spikelet pair meristem; SM, spikelet meristem; FM-S1, floral meristem at stage 1; FM-S2, visible floral organs at stage 2; FM-S3, sexual organs being suppressed at stage 3. B. High-confidence co-expressed network of Zm00001eb194930, Zm00001eb159640, Zm00001eb195380, Zm00001eb352910, Zm00001eb429430, Zm00001eb429900, Zm00001eb430240(*zfl1*). Dots represent genes.
